# Hybrid Hyaluronic Acid versus High Molecular Weight Hyaluronic Acid for the Treatment of Hip Osteoarthritis in Overweight/Obese Patients

**DOI:** 10.3390/jfmk7010020

**Published:** 2022-02-09

**Authors:** Dalila Scaturro, Fabio Vitagliani, Pietro Terrana, Sofia Tomasello, Vincenzo Falco, Daniele Cuntrera, Italia Spoto, Massimo Midiri, Giulia Letizia Mauro

**Affiliations:** 1 Department of Surgical, Oncological and Stomatological Disciplines, University of Palermo, 90100 Palermo, Italy; dalila.scaturro@unipa.it (D.S.); giulia.letiziamauro@unipa.it (G.L.M.); 2 Faculty of Medicine and Surgery, University of Catania, 95100 Catania, Italy; 3 Faculty of Medicine and Surgery, University of Palermo, 90100 Palermo, Italy; terranapietro@gmail.com (P.T.); sofia.tomasello@gmail.com (S.T.); 4 Department of Economics and Statistics, University of Palermo, 90100 Palermo, Italy; vincenzo.falco01@unipa.it (V.F.); daniele.cuntrera@unipa.it (D.C.); 5 Department of Biomedicine, Neuromedicine and Advanced Diagnostics, University of Palermo, 90100 Palermo, Italy; italia.spoto@unipa.it (I.S.); massimo.midiri@unipa.it (M.M.)

**Keywords:** obesity, hip osteoarthritis, viscosupplementation, rehabilitation

## Abstract

Background: Obesity is the main risk factor for hip osteoarthritis, negatively affecting the outcome of the disease. We evaluated the effectiveness of viscosupplementation with hybrid hyaluronic acid compared to that with high molecular weight hyaluronic acid in overweight/obese patients with hip osteoarthritis (OA). Methods: 80 patients were divided into two groups: a treatment group received two ultrasound-guided intra-articular hip injections of hybrid HA 15 days apart; a control group received a single ultrasound-guided infiltration with medium-high molecular weight hyaluronic acid (1500–2000 kDa). We assessed the pain, functional and cardiovascular capacity of the patients at baseline, after 3 months, and after 6 months of the infiltrative sessions. Results: The treatment group showed greater improvements in the scores on the NRS scale (5.4 ± 0.8 vs. 6.3 ± 0.8; *p* < 0.05) and in the Lequesne index (11.4 ± 2.6 vs. 13.6 ± 2.7; *p* < 0.05) and in the distance traveled at 6MWT (238.1 ± 53.9 m vs. 210.7 ± 46.2 m; *p* = 0.02) both at 3 months (T1) and at 6 months (T2). Conclusions: This study underlines the importance of exploiting the anti-inflammatory, analgesic, and chondrogenic properties of hybrid HA for the treatment of hip OA in overweight/obese patients.

## 1. Introduction

Hip OA is a chronic musculoskeletal disease that is widespread and causes disability worldwide, with one in four people developing it by the age of 85 [1]. The predominant symptoms are pain and stiffness, typically leading to loss of physical function, impaired walking, and reduced quality of life. From a structural point of view, it is characterized by the loss of articular cartilage and by alterations of the subchondral bone in terms of turnover, mineralization, and volume [2]. In advanced hip osteoarthritis, muscles are also affected and atrophic muscle weakness in the lower limbs is likely to be the cause of the reported reduced physical function in these patients [3].

Damage due to mechanical overload is mainly recognized in the genesis of osteoarthritis [4]. Among the risk factors for OA, a positive correlation between high body mass index (BMI) and hip OA is now known [5,6]. Although this correlation is less strong than that with osteoarthritis of the knee, a recent meta-analysis found an 11% increase in the risk of hip OA per five-unit increase in BMI in both sexes [7]. The mechanisms linking BMI increase to hip OA pathogenesis are twofold [8]. On the one hand, excess body weight increases the biomechanical load on the hip joint, contributing to joint damage. On the other hand, obesity affects the metabolic environment of the joint due to the pro-inflammatory systemic activation associated with obesity, increasing the risk of hip OA [9,10].

Obesity in patients with hip OA is responsible for an increase in health care costs and negatively affects the outcome of the disease, such as the need to resort to surgical arthroplasty [10]. The American Association of Hip and Knee Surgery (AAHKS) recommends delaying the use of total arthroplasty in patients with BMI ≥ 40 kg/m^2^, especially in association with other comorbidities (e.g., diabetes) [10], as it is associated with an increased risk of post-surgical complications. These include a higher risk of infection, early failure requiring revision, worse functional outcomes, and increased use of health care. [10]. Furthermore, obese patients undergoing hip arthroplasty have a greater risk of having worse outcomes in terms of pain and complications than patients of normal weight [11].

In obese patients with hip OA, conservative treatment is always recommended and preferable. Weight reduction is the first recommendation followed by rehabilitation treatment [12]. Treatment goals for hip OA are pain relief, reduction of swelling and stiffness, improvement of mobility and quality of life, and slowing of disease progression [13,14].

According to recent guidelines from the American College of Rheumatology, exercise, aimed at strengthening muscles and improving range of motion, and weight loss are recommended in patients with OA of the hip. A loss of ≥5% of body weight promotes noticeable changes in clinical outcome, with these benefits continuing to increase with further weight loss [10,15].

Among the options for the pharmacological treatment of OA, paracetamol, non-steroidal anti-inflammatory drugs (NSAIDs), and cycle-oxygenase 2 inhibitors (COX-2) are commonly prescribed; however, due to the patient’s poor compliance and their gastrointestinal, cardiovascular, and renal toxicity, intra-articular viscosupplementation with hyaluronic acid is an alternative for patients who are intolerant to oral and topical analgesics. [16].

Viscosupplementation is a non-drug and minimally invasive treatment option for OA. The intra-articular administration of preparations based on hyaluronic acid (HA), the main structural and biochemical molecule of cartilage, was approved in 1997 by the Food Drug Administration (FDA), to integrate synovial fluid in the affected joint. [17]. By effectively increasing viscosity and reducing friction, viscosupplementation with hyaluronic acid (HA) has been shown to achieve satisfactory pain relief and improved function in OA [18,19,20]. Furthermore, HA is known to mitigate the activity of pro-inflammatory mediators and stimulate the metabolism of fibroblasts, possibly resulting in analgesic, anti-inflammatory, and chondroprotective effects on the joint [21].

Many HA products on the market differ based on: different sources of HA, structure, molecular weight, concentration, volume per injection, and a number of injections per course of therapy. Based on the molecular weight, the different products available are divided into three categories: low molecular weight (LMW-HA) (MW: 0.5–1.5 million Dalton); medium molecular weight (MMW-HA) (MW: 1.5–6 million Dalton); high molecular weight (HMW-HA) (MW: 6–7 million Dalton). Many studies state that high molecular weight intra-articular hyaluronic acids (HMW-HA) have better chondro-protective, anti-inflammatory, proteoglycan, rheological, analgesic, and mechanical properties [16].

Sinovial^^®^^ HL by IBSA is a formulation of stable and co-operative hybrid HA complexes produced by a patented thermal process from a combination of high (1100–1400 kDa) and low (80–100 kDa) molecular weight (MW) hyaluronans, without the need for 1,4-butanediol diglycidyl ether (BDDE) or other chemicals. The unique characteristics of Sinovial^^®^^ H-L include a high concentration of HA (64 mg in 2 mL), low viscosity with optimal tissue diffusion, and durability comparable to a weakly cross-linked gel. In mild to moderate hip OA, intra-articular injections of Sinovial^^®^^ are effective in improving pain and function, without serious adverse events [22].

The purpose of the study was to evaluate the effectiveness of viscosupplementation with hybrid hyaluronic acid against viscosupplementation with high molecular weight hyaluronic acid in overweight/obese patients with II–III degree according to K–L hip osteoarthritis.

## 2. Materials and Methods

It is a randomized single-center, case-control clinical study conducted during the period between January 2020 and April 2021 on patients treated for symptomatic OA of the hip with hyaluronic acid infiltration, at the rehabilitation department of the Paolo Giaccone Hospital Policlinico Universitario of Palermo.

The study was conducted following the ethical guidelines of the Declaration of Helsinki; the local ethics committee “Palermo 1” approved the study, with the reference number 11/2020; the information and data were managed according to the guidelines of the Good Clinical Practice (GCP). All participants signed an informed consent form at the time of enrollment to collect clinical data. All patients also gave their consent to the adoption of evaluation questionnaires.

The inclusion criteria were: age ≥ 30 years; BMI ≥ 25 kg/m^2^; radiographic evidence of coxarthrosis II-III degree according to K-L; no previous infiltrative treatment in the previous 6 months; written consent for participation in the study.

The exclusion criteria were: secondary OA (after acetabular or cephalic fracture, avascular necrosis, developmental dysplasia, slipped capital femoral epiphysis, Legg–Calvè-Perthes disease, primary inflammatory rheumatic diseases), altered states of consciousness, neurological, musculoskeletal dysfunctions, and cardiorespiratory.

The recruited patients were randomly divided, using a computer-generated random number table, into two groups: the treatment group, consisting of 43 patients who received two ultrasound-guided intra-articular injections of hybrid HA complexes (64 mg/2 mL) (Sinovial^^®^^ HL) into the joint hip 15 days apart; and the control group, consisting of 37 who received a single ultrasound-guided infiltration with medium-high molecular weight hyaluronic acid (60 mg/4 mL) (1500–2000 kDa) in the hip joint.

All patients underwent a rehabilitation program of 20 sessions every three weeks lasting 60 mins and consisting of functional re-education with exercises to strengthen the quadriceps femoris muscle, stretching of the hamstrings, and proprioceptive exercises. Recruited patients did not receive anti-inflammatory or pain-relieving drugs in the week before the first injection.

Patients were evaluated at three time points: at baseline (T0), at 3 months (T1) and at 6 months (T2). At each evaluation, demographic information and clinical data relating to the status of the disease were collected.

To evaluate the effect of HA viscosupplementation in patients with hip OA, we evaluated the following primary endpoints: pain, with the NRS scale [23]; functional residual capacity (cardio-pulmonary), with the Six-Minute-Walking-Test (6MWT) [24]; severity of the disease, with the Lequesne index [25]. In addition to these primary endpoints, we also considered four secondary endpoints: quality of life, as assessed by the SF-12 scale [26]; the percentage of fat and lean mass, measured by analysis of the bioelectrical impedance; taking analgesics, expressed as the number of days per week it takes to achieve pain control. Finally, we also recorded adverse reactions to treatment.

The NRS scale is a quantitative rating scale by which patients are asked to rate their pain on a defined scale, from 0 to 10 [23].

The 6MWT is a simple method used to assess the physical performance of patients in many different disease groups. It consists of the measurement of the distance traveled on foot in 6 min. During this test, data can also be collected via blood oxygen saturation and the patient’s heart rate, and the possible perception of dyspnea from exertion [24].

The Lequesne Algofunctional Index is an 11-question survey developed to assess physical function in OA: five questions relate to pain and discomfort, two to maximum distance traveled, and four to activities of daily living. The overall score is 0–24 points [25].

The 12-Item Short Form Survey (SF-12) scale is the abbreviated version of the Short Form 36 items Health Survey (SF36) questionnaire and serves as a generic indicator of the quality of life. It consists of 12 questions that investigate eight different health aspects: physical activity; role limitations due to physical health; emotional state; physical pain; perception of general health; vitality; social activities; and mental health. The lower the score, the greater the degree of disability [26].

For the ultrasound-guided infiltration method, a sterile convex probe with a 20 G guide and Power-Doppler was used to visualize the blood vessels and avoid perforation. The patient was positioned supine with the hip in an internal rotation of 15–20°. We used an anterior parasagittal approach. With the aid of a biopsy guide, a 20 G (9 cm) spinal needle was inserted with an anterior-superior approach to perform the intra-articular injection. The needle was advanced into the anterior capsular recess, stopping approximately 1 mm from the femoral head. HA was then injected into the hip joint, verifying intra-articular positioning in real-time via US (direct visualization of viscous fluid or air bubbles) [27].

### Statistic Analysis

All analyzes are performed using R software (R Core Team, 2013; Vienna, Austria). For the calculation of the sample size, we used the following formula:

n=zα22σ2ε2=zα22π1−πε2 n=1.962*0.5*0.50.11^2=79.37≈80.

Since no pilot study was conducted to estimate *σ*^2^, we took the conservative assumption (i.e., variance of 0.5) into account and set the maximum permissible error at 0.11. Thus, we obtained that the sample size must be 79.27. For the statistical test, we used three different tests: the *t*-test to compare means for quantitative variables, the Mood’s median test to compare medians for ordinal variables, and the test for two proportions. The results were considered statistically significant with a *p*-value < 0.05.

## 3. Results

A total of 80 patients with coxarthrosis met the inclusion criteria and were deemed eligible to participate in this study. The general characteristics at baseline of the population under examination, 52 females (65%) and 28 males (35%) with mean age of 66.1 ± 9.7 years, are shown in Table 1.

No statistically significant differences were noted between the two groups at baseline between the collected data. However, all patients completed the proposed in-filtration cycle. Five patients in the treatment group and three patients in the control group reported mild and transient adverse events, predominantly represented by local pain at the injection site and a sensation of joint encumbrance, while no patient reported severe adverse events at the end of treatment.

Table 2 shows the changes in the values of the primary endpoints at T1 and T2 in the two groups under consideration. At three months from the infiltrative treatment (T1) in the treatment group, we observed a greater improvement, compared to the control group, for the mean scores of the NRS scale (5.4 ± 0.8 vs. 6.3 ± 0.8; *p* < 0.05) and in the Lequesne (11.4 ± 2.6 vs. 13.6 ± 2.7; *p* < 0.05). The gain in terms of distance traveled at 6MWT was also higher in the treatment group than in the control group (238.1 ± 53.9 m vs. 210.7 ± 46.2 m; *p* = 0.02) (Table 2). In the follow-up 6 months after the infiltrative treatment (T2) the mean scores of the NRS scale and the Lequesne index in the treatment group worsened slightly, remaining however lower than those observed in the control group (NRS scale:5.9 ± 0.9 vs. 6.8 ± 1.1; *p* < 0.05) (Lequesne index: 11.9 ± 2.2 vs. 14 ± 2; *p* < 0.05). Finally, the recovery of residual functional capacity also remained higher in the treatment group compared to the control group in the follow-up at 6 months (225.4 ± 45.5 m vs. 204.8 ± 35.8 m; *p* = 0.03) (Table 2).

Analyzing the variation at 3 months (Δ(T1-T0)) and at 6 months (Δ(T2-T0)) of the primary endpoint scores in the two groups, we observed that in both follow-up periods, the treatment group showed statistically significant changes compared to the treatment group, in scores of the NRS scale (Δ(T1-T0) *p* = 0.01; Δ(T2-T0) *p* < 0.01), Lequesne index (Δ(T1-T0) *p* < 0.01; Δ(T2-T0) *p* < 0.01) and in the distance traveled at 6MWT (Δ(T1-T0) *p* = 0.01; Δ(T2-T0) *p* < 0.01) (Table 3).

Table 4 shows the changes in the secondary endpoints at T1 and T2 in the two groups under examination. None of the analyzed variables show statistically significant improvements. In both groups, there is an increase in the SF-12 score in the 3-month follow-up (27.7 ± 5.0 vs. 25.9 ± 5.2; *p* = 0.12), while a slight decrease in the 6-month follow-up is observed (26.9 ± 4.6 vs. 25.2 ± 3.9; *p* = 0.08) while remaining higher than baseline. In the treatment group we observed a slight increase in muscle mass at T1 (39.1 ± 6.7 vs. 37.4 ± 5.2; *p* = 0.21) and at T2 (39.9 ± 6.7 vs. 37.9 ± 4.8; *p* = 0.13), while the fat mass remained almost the same in both the treatment group and the control group at T1 (35.4 ± 8.7 vs. 35.7 ± 8; *p* = 0.87) and T2 (35.2 ± 8.7 vs. 35.7 ± 7.7; *p* = 0.78) (Table 5).

Regarding the average consumption of analgesics, 88.4% of patients took analgesics more than twice a week at T0 in the treatment group, while at T1 46.5% and finally at T2 62.8%. In the control group, on the other hand, at T0 93.1% of patients took analgesics more than twice a week, while at T1 44.2%, and finally at T2 34.9%.

Table 5 shows the variations (T1-T0) and (T2-T0) of the secondary endpoints in the two groups under examination. Despite a greater improvement in the SF-12 scale scores in the treatment group compared to the control group, no statistically significant difference was seen in the (T1-T0) and (T2-T0) variations between the two groups (Δ(T1-T0) *p* = 0.09; Δ(T2-T0) *p* = 0.13). Finally, no statistically significant difference was observed regarding the (T1-T0) and (T2-T0) changes in muscle mass (Δ(T1-T0) *p* = 0.94; Δ(T2-T0) *p* = 0.25) and in the fat mass (Δ(T1-T0) *p* = 0.20; Δ(T2-T0) *p* = 0.62).

## 4. Discussion

In obese patients, conservative management of hip OA with Hyaluronic Acid (HA) is of fundamental importance to reduce pain and improve quality of life, avoiding surgery as much as possible and reducing any risks or complications [10].

HA is widely used for knee OA, however, only a few studies investigate its use in hip OA [19,28]. Although HA infiltrative therapy is the best conservative therapy before surgery, as it acts on pain without modifying the anatomical structure of the hip, there are no guidelines on the ideal number of injections needed to achieve an optimal clinical response, which appears to be different [1,2,13,15,17,28] between products and dependent on molecular weight (MW) [8].

Generally, one to four injections are recommended with the ultrasound-guided infiltration technique, which is the safest approach [28,29,30].

In the course of this study, we made a comparison between the use of a double joint infiltration with hybrid HA in the treatment of hip osteoarthritis compared to a single intra-articular infiltration with medium-high molecular weight HA. The goal was to establish an infiltrative protocol based on the use of hybrid HA to confirm the efficacy of non-surgical treatment of hip OA in obese patients. In this study, overweight and obese patients who came to our clinics were divided into two groups, a first group undergoing two intra-articular injections of hybrid HA 14 days apart and a second group undergoing a single injection of HA high molecular weight. In both groups, we observed an improvement in pain related to hip osteoarthritis, in the functional and cardio-circulatory capacity of the patients in the 3-month follow-up. Furthermore, all these results were maintained, support to a lesser extent, in the follow-up 6 months after the infiltrative treatment.

From the proposed data, we observed that these improvements proved to be superior in the group of patients who received double intra-articular infiltration with hybrid hyaluronic acid compared to those who received single infiltration with medium-high molecular weight hyaluronic acid.

Our results are in line with other studies in the literature that have evaluated the efficacy of hybrid HA in the treatment of OA [31,32,33]. The reasons for these results could derive from the characteristics of HA [29,31,32]. HA has a dual action of viscosupplementation, linked to its viscosity (short-term effect), and biosupplementation, linked to its chondroprotection and stimulation of the endogenous synthesis of HA (long-term effect). Papalia et al. [31], compared the effectiveness of viscosupplementation with hybrid HA compared to that with high molecular weight HA in obese patients with osteoarthritis of the knee. The authors observed better outcomes in patients treated with hybrid HA than in those treated with high molecular weight HA, both in terms of pain and knee function. Abate et al. [32], observed that the administration of hybrid HA, made from a combination of LMW and HMW HA, is safe and effective in the treatment of moderate-severe hip OA and guarantees superior results compared to HA. HMW. The authors attributed this greater efficacy to the fact that low molecular weight compounds have a greater biosupplementation action, while high molecular weight ones have a greater viscosupplementation action. The administration of a hybrid HA, made from a combination of low and high-molecular-weight hyaluronates, cooperating with each other, seems to offer better results. HMW HA through its hydrophilic properties and the increase of the residence time in the joint improves the viscoelastic properties (viscosupplementation), while the greater penetration of LMW HA into the extracellular matrix of the synovium positively influences various biological activities [32].

Finally, in our study, we also observed a reduction in the number of days of analgesics per week and an improvement in the quality of life perceived by patients, although these parameters did not reach statistical significance in either group.

The reduction in the consumption of analgesics was particularly important in consideration of the numerous side effects associated with the improper use of NSAIDs, especially in the elderly and overweight population prone to OA. These data are in line with the data in the literature [34] and indicates that HA injections reduce the intake of NSAIDs/analgesics, avoiding the most frequent complications related to these drugs (e.g., gastrointestinal/renal complications and increased cardiovascular mortality) [35,36].

In the literature, numerous studies have been conducted to evaluate the effectiveness of the various HA compounds in knee osteoarthritis, while only a few studies have been conducted to compare the effectiveness of the different types of HA in the conservative management of hip osteoarthritis [30,37,38,39].

Van den Bekerom et al. [30], evaluated the efficacy of various medium molecular weight HA compounds in the treatment of hip osteoarthritis in patient candidates for hip arthroplasty. After six weeks from the end of the infiltrative treatment, all patients showed a reduction in pain and an improvement in function, assessed by the Harris Hip Score, without, however, observing statistically significant differences between the three compounds. Following the treatment, the authors also noted that only 51% of patients, after three years, did not undergo surgery.

Rivera et al. [37] conducted a study on 59 patients with hip osteoarthritis comparing the effect of high molecular weight HA and low molecular weight HA. The authors concluded that a single dose of high molecular weight HA is effective for pain control in patients with hip arthritis, especially from the third month onwards, with results remaining stable or continuing to improve until about 1 year.

Tikiz [38] compared the effectiveness of intra-articular injections of an LMW-HA (Ostenil, 1.2–1.4 × 106 DA) with a higher molecular weight viscosupplement (Hylan GF 20, Synvisc, 7 × 106 DA) in hip arthrosis. The authors observed a significant reduction in pain on the VAS scale and in WOMAC and Lequesne scores in both groups for up to six months, without however observing significant differences between the two groups.

Clementi et al. [39] conducted a double-blind prospective comparative study to compare the efficacy of intra-articular injections of a very high molecular weight viscosupplement with a medium molecular weight hyaluronic acid in hip OA. A single dose of very high molecular weight hyaluronic acid resulted in clinical effects on pain and disability compared to those obtained with two doses of low molecular weight hyaluronic acid in the OA of the hip.

To our knowledge, our study was the first to have compared the effectiveness of viscosupplementation with hybrid HA compared to that with medium-high molecular weight HA, in an overweight/obese population with hip osteoarthritis.

Taken together, the results are very encouraging in terms of the ability of HA hybrid viscosupplementation to relieve pain, curb disability and limit the risk of polypharmacy in overweight patients.

However, our study has some limitations, mainly represented by the low number of the sample, deriving from the short period of the research, which does not allow us to generalize the results obtained. A further limitation is the lack of a long-term follow-up that would have made it possible to detect further important information, namely the time interval for a new infiltrative cycle.

## 5. Conclusions

In conclusion, the present study adds to the growing evidence of the importance of exploiting the anti-inflammatory, analgesic, and chondrogenic properties of hybrid HA for the treatment of hip OA. It also provides important new data on the safety and efficacy of this non-invasive, non-drug treatment option in overweight and obese patients. Finally, the US-guided viscosupplementation technique was found to be safe and well-tolerated by patients.

## Figures and Tables

**Table 1:** General characteristics at baseline.

Characteristics	Total (*n* = 80)	Treatment Group (*n* = 43)	Control Group (*n* = 37)	*p*-Value
Age, mean ± SD	66.1 ± 9.7	65.2 ± 10.3	67.1 ± 9.0	0.38
Sex, n° (%) Female Male	52 (65) 28 (35)	32 (74) 11 (26)	20 (54) 17 (46)	0.11
BMI (Kg/m^2)^, mean ± SD	29.9 ± 4.6	29.5 ± 4.5	30.3 ± 4.7	0.47
Side, n° (%) Right Left	47 (58.8) 33 (41.2)	25 (58.1) 18 (41.9)	22 (59.5) 15 (40.5)	0.24
Fat mass (%), mean ± SD	35.6 ± 8.3	35.5 ± 8.7	35.8 ± 7.9	0.87
Muscle mass (%), mean ± SD	37.7 ± 6.1	38.4 ± 6.7	36.8 ± 5.2	0.24
NRS, mean ± SD	7.2 ± 0.8	7.3 ± 0.8	7.1 ± 0.9	0.99
Lequesne scale, mean ± SD	14.4 ± 2.8	14.4 ± 2.9	14.3 ± 2.7	0.87
SF-12 scale, mean ± SD	25 ± 5.1	25.2 ± 5.2	24.8 ± 4.9	0.72

**Table 2:** Primary endpoints at 3 months (T1) and 6 months (T2).

Characteristics	T0 (Baseline)	T1 (3 Months)	T2 (6 Months)
NRS, mean ± SD			
GT	7.2 ± 0.8	5.4 ± 0.8	5.9 ± 0.9
CG	7.1 ± 0.9	6.3 ± 0.8	6.8 ± 1.1
*p*-value	0.99	<0.05	<0.05
Lequesne Index, mean ± SD			
GT	14.4 ± 2.9	11.4 ± 2.6	11.9 ± 2.2
CG	14.3 ± 2.7	13.6 ± 2.7	14 ± 2
*p*-value	0.87	<0.05	<0.05
Distance traveled (meters), mean ± SD			
TG	189.2 ± 56.7	238.1 ± 53.9	225.4 ± 45.5
CG	192 ± 50.9	210.7 ± 46.2	204.8 ± 35.8
*p*-value	0.82	0.02	0.03

**Table 3:** Primary endpoint variation at 3 months (Δ(T1-T0) and at 6 months (Δ(T2-T0)).

Characteristics	ΔT1-T0	ΔT2-T0
NRS, median		
Treatment Group	−2 (1)	−1 (1)
Control Group	−1 (1)	0 (2)
*p*-value	0.01	<0.01
		
Lequesne Scale, median		
Treatment Group	−3 (2)	−3 (2.5)
Control Group	−1 (1)	0 (1)
*p*-value	<0.01	<0.01
Distance traveled (meters), mean ± SD		
Treatment Group	48.9 ± 38.8	36.2 ± 62.0
Control Group	18.7 ± 17.1	12.8 ± 39.3
*p*-value	<0.01	<0.01

**Table 4:** Secondary endpoint at 3 months (T1) and 6 months (T2).

Characteristics	T0 (Baseline)	T1 (at 3 Months)	T2 (at 6 Months)
SF-12 scale, mean ± SD			
Treatment Group	25.2 ± 5.3	27.7 ± 5.0	26.9 ± 4.6
Control Group	24.8 ± 4.9	25.9 ± 5.2	25.2 ± 3.9
*p*-value	0.72	0.12	0.08
Muscle Mass (%), mean ± SD			
Treatment Group	38.4 ± 6.8	39.1 ± 6.7	39.9 ± 6.7
Control Group	36.8 ± 5.3	37.4 ± 5.2	37.9 ± 4.8
*p*-value	0.25	0.21	0.13
Fat Mass (%), mean ± SD			
Treatment Group	35.5 ± 8.8	35.4 ± 8.7	35.2 ± 8.7
Control Group	35.8 ± 8.1	35.7 ± 8.0	35.7 ± 7.7
*p*-value	0.87	0.87	0.78

**Table 5:** Secondary endpoint variation at 3 months (Δ(T1-T0)) and at 6 months (Δ(T2-T0)).

Characteristics	ΔT1-T0	ΔT2-T0
SF-12 scale, mean ± SD		
Treatment Group	2.5 ± 4.9	1.8 ± 4.9
Control Group	1.1 ± 2.4	0.4 ± 2.8
*p*-value	0.09	0.13
Muscle Mass (%), mean ± SD		
Treatment Group	−0.1 ± 0.6	1.4 ± 0.9
Control Group	−0.1 ± 0.7	1.1 ± 1.2
*p*-value	0.94	0.25
Fat Mass (%), mean ± SD		
Treatment Group	−1.4 ± 0.9	−0.3 ± 1.5
Control Group	0.5 ± 0.8	−0.1 ± 1.6
*p*-value	0.20	0.62

## Data Availability

Data used to support the findings of this study are available from the corresponding author upon request.
